# Adapting the Quality Maternal and Newborn Care (QMNC) Framework to evaluate models of antenatal care: A pilot study

**DOI:** 10.1371/journal.pone.0200640

**Published:** 2018-08-14

**Authors:** Andrew Symon, Alison McFadden, Marianne White, Katrina Fraser, Allison Cummins

**Affiliations:** 1 Mother and Infant Research Unit, School of Nursing and Health Sciences, University of Dundee, Dundee, United Kingdom; 2 Maternity Services, Ninewells Hospital, NHS Tayside, Dundee, United Kingdom; 3 Maternity Unit, Victoria Hospital, NHS Fife, Kirkcaldy, United Kingdom; 4 Centre for Midwifery, Child and Family Health, University of Technology Sydney, Sydney, Australia; TNO, NETHERLANDS

## Abstract

**Background:**

Recent evidence indicates that continuity models of maternity care result in improved clinical and psychosocial outcomes, but their causal mechanisms are poorly understood. The recent Lancet Series on Midwifery’s Quality Maternal and Newborn Care Framework describes five components of quality care and their associated characteristics. As an initial step in developing this Framework into an evaluation toolkit, we transformed its components and characteristics into a topic guide to assess stakeholder perceptions and experiences of care provided and received. The main purpose of this study was to assess the feasibility of this process.

**Methods:**

We conducted twelve focus groups in two Scottish health board areas with 13 pregnant women, 18 new mothers, 26 midwives and 12 obstetricians who had experience of a range of different models of maternity care. Transcripts were analysed using a six-phase approach of thematic analysis. We mapped the identified themes and sub-themes back to the Framework.

**Results:**

The emerging themes and sub-themes demonstrated the feasibility of using the QMNC framework as a data collection tool, and as a lens for analysing the data. Of the four emerging themes, only Organisation Culture / Work Structure’ mapped directly to a single Framework component. The others—‘Relationships’; ‘Information and support’; and ‘Uncertainty’–mapped to between two and five components, illustrating the interconnectedness of the Framework’s components. Some negative sub-themes mirrored positive Framework characteristics of care. Some re-phrasing and re-ordering of the topic guides in later focus groups ensured we could cover all aspects of the Framework adequately.

**Conclusion:**

Adapting the Quality Maternal and Newborn Care Framework enabled us to focus on aspects of care which worked well and which didn’t work well for these key stakeholders. Identifying ‘what works for whom and why’ in different models of care is a necessary step in reinforcing and replicating the most effective models of care.

## Introduction

Delivering high quality maternity care is a key policy driver in the UK, following recent government reviews of maternity services in England (*Better Births*) [1] and Scotland (*The Best Start*) [2]. These were produced against a backdrop of serious failures of clinical maternity care in one NHS organisation in the UK [3], and the fact that the UK fares worse than some comparable countries in key perinatal outcomes, e.g. preterm birth [4], and perinatal mortality [5]. These outcomes entail significant clinical, psychosocial, organisational and financial burdens.

A considerable influence on policy development has been the growing evidence concerning the benefits of continuity of care. Sandall et al’s meta-analysis of trials in their Cochrane review demonstrated that midwife-led continuity of care reduces the likelihood of preterm birth and the incidence of clinical interventions, as well as contributing to improved psychosocial outcomes [6]. While trial reports seldom explain the model’s active mechanisms, i.e. how, where and why care is given and which aspects make a difference [7], it is reasonable to assume that better outcomes result from better quality care. Understanding which aspects of care make the difference is crucial to replicating and implementing cost-effective high quality care.

A major breakthrough in understanding the constituent elements of quality maternity care came in 2014 with the publication of the Quality Maternal and Newborn Care (QMNC) Framework [‘the Framework’] in The Lancet Series on Midwifery [8]. The image cannot be reproduced here, but is available online (http://bit.ly). Publication of the Framework followed an extensive analysis and synthesis of the global literature by an international group of researchers. Organised into five components (Practice categories; Organisation of care; Values; Philosophy; Care providers) which each list relevant characteristics of quality care, the Framework provides a detailed overview of what constitutes good quality maternal and newborn care. In theory, this then provides a benchmark against which (as well as a ‘lens’ by which) to evaluate service provision. The Framework’s authors assert that while the two boxes in the ‘top right corner’ focus on the characteristics of care needed for ‘childbearing women and infants with complications’, all childbearing women and infants should receive all the other care described in the remaining framework components.

The Framework has been proposed as a structure around which improvements in midwifery can be made globally [9]. It has been used to inform the redesign of a midwifery curriculum in Australia [10] as well as revised international benchmarks for antenatal care [11], and was influential in producing *The Best Start* [2] in Scotland. However, while the Framework describes the best available evidence for quality maternal and newborn care, it is not known how existing antenatal, intrapartum and postnatal services match up to this benchmark, or what helps or hinders the implementation or extension of successful models. The aim of this exploratory study was to assess the feasibility of developing the Framework into a topic guide to assess maternity care stakeholder perceptions and experiences, this being the first step in developing an evaluation toolkit for maternity care. Through focus groups involving service users (pregnant women and new mothers) and service providers (midwives and obstetricians), we explored perceptions of receiving or providing maternity care, and whether this reflected the characteristics of good quality care as detailed in the Framework. Ascertaining what works and what doesn’t is necessary if policy-makers and service planners are to identify what aspects of care delivery to reinforce or replicate. It should also help focus attention on those areas which need to be addressed.

## Materials and methods

As part of a research capacity-building exercise, two midwives (MW, KF: one from each of two Scottish Health Board [HB] areas) were seconded to the Mother and Infant Research Unit, University of Dundee, as Research Assistants (RAs) to help facilitate, document and analyse the focus group discussions. Training from within the university was provided, including facilitating and analysing a ‘practice’ focus group with post-graduate students; this allowed the RAs to practise their facilitation and note-taking skills within the focus group context. A researcher (AC) from Australia with experience in qualitative research into midwifery models of care also collaborated in the data generation and analysis. AS conducted eleven focus groups with MW (n = 6) and KF (n = 5); AM conducted one focus group with MW; AC attended two focus groups. All five team members are midwives.

Focus group topic guides were derived through a process of distilling the described characteristics of care from the Framework into a usable format (see ‘topic guide’ supplementary file). AM was one of the co-authors of the article introducing the Framework, and the lead author from that article was also consulted. For each of the Framework’s five components we devised a principal question together with supplementary prompts. For example, for the component ‘Practice categories’ we initially asked service users “Do you feel the model of care you have experienced covers all the necessary bases?” Providers were asked “Do you feel the model of care in your current workplace covers all the necessary bases?” Supplementary prompts referred to health promotion, screening, care planning, *etc*., as detailed in the Framework. As part of our on-going assessment of the most effective way to stimulate relevant discussion, we re-framed and re-ordered some questions.

### Analysis

Data were analysed by a six-phase approach of thematic analysis [12]; we did not use data management software. The RAs (MW and KF), who kept reflective diaries so that their experience of helping to organise, run and analyse the focus groups would help to provide insights into the success or otherwise of our chosen approach, transcribed five interviews; a professional transcription service was used for the remainder. Phase One of the analysis involved familiarisation with the data through repeated reading by the relevant RA and at least one other member of the team; this process enabled the team members to become immersed in the data, searching for meaning and patterns. To enhance the credibility of the interpretation we paid attention to negative and dissonant cases.

In Phase Two, the data were organised into initial codes. Phase three of the analysis involved sorting all the codes into themes and a provisional coding frame; this was derived both deductively (using constructs from the Framework) and inductively (incorporating new themes that emerged from the data through open coding). This provisional analysis was then read and reread by other members of the research team, and the codes and themes discussed and debated. This process of reading, rereading and ensuring all members of the team agreed on the codes and emerging themes ensured rigour in the analysis process. This approach enabled comparison by themes across different focus groups as well as retaining the context of individual experiences. We sought to assess how well (and in what way) the emerging themes and sub-themes reflected the Framework, and how much was distinct from it. We referred to the tone of these sub-themes as being ‘positive’ or ‘negative’.

The fourth phase involved reviewing the themes and the positive and negative sub-themes. The Framework helped to shape the final themes and sub-themes arrived at in Phase 5. The sixth phase of data analysis was the final write up, embedding data extracts within the analysis.

#### ‘Top 4’ sub-themes

In order to help focus our analysis and find areas of commonality and difference between the groups we identified the four principal topics of discussion within each group. We refer to these as the focus group’s ‘Top 4’ sub-themes. While we refer to these commonly-discussed sub-themes in our findings here, a separate paper will examine the commonalities and differences between different models of care in depth.

### Participants and recruitment

We initially planned to hold ten focus groups. Service users were recruited purposively to include both antenatal and postnatal women with experience of different types of care provision. MW and KF liaised with local community-based or maternity unit-based midwives, health visitors and administrative staff to identify potential participants from midwifery and obstetric clinic lists. Advertising posters were also left prominently in the relevant clinics. Those eligible were women in the third trimester of pregnancy who had experience of one of the local antenatal models of care, and new mothers (with babies up to five months of age) who had recently experienced one of these models of care. Women who were deemed unable to understand the nature of the study (either through language barrier or cognitive impairment), or who were either emotionally and/or physically seriously unwell, were not eligible. Likewise, women whose babies had died or who were seriously unwell, and those under the age of 16, were not approached. Potential participants were sent an invitation letter from the local Head of Midwifery and a Participant Information Sheet (PIS) which explained who the researchers were and why they were conducting this study. They were invited to contact the relevant Research Assistant (RA); alternatively, they could leave a reply slip at the antenatal clinic for the RA to collect, or give this to their midwife or health visitor to forward to the RA. Those participating received a £10 ‘thank you’ shopping voucher.

Service providers were purposively approached, to obtain a range of views from both senior and junior practitioners. One midwives’ group was specifically for newly qualified midwives, *i*.*e*. those practising within one year of registration. However, midwives and obstetricians of any grade working in the two HB areas were eligible for the other service provider focus groups. We used open advertisement, with posters in prominent areas, so that others who wished to participate could contact the study team. All staff members doing so, and all those purposively selected, were sent an invitation letter and PIS. All potential participants who indicated preparedness to attend a focus group were advised of possible dates, times and venue.

We conducted twelve focus groups involving 13 pregnant women, 18 new mothers, 26 midwives and 12 obstetricians, who between them had experience of a range of different models of care (Table 1). These models are based essentially on whether they are provided for all women, irrespective of their risk level (which we term ‘Universal Provision model’), or whether they are targeted at specific groups of women, often based on their assessed risk status. The ‘Higher risk model’ is for women with identified risk factors (which can be obstetric, medical, or social). The ‘Case loading model’ refers to an arrangement whereby an individual midwife is the primary care giver for a specified number of women, usually ‘low risk’, following them throughout the childbirth continuum. The locations for the care given vary according to local circumstances. In this study, they ranged from the community (either the woman’s home or a health centre), to community maternity units (often called ‘midwife-led units’) and tertiary referral hospitals.

**Table 1:** Focus group participants and principal type(s) of care model.

Focus group	Participants	Focus of care / type of model experienced
**#3**	Pregnant women	Higher risk model
**#6**	Pregnant women	Higher risk model
**#11**	Pregnant women	Universal Provision model
**#7**	Pregnant women and new mothers	Case loading model
**#5**	New mothers	Universal Provision model
**#8**	New mothers	Universal Provision model
**#10**	New mothers	Universal Provision model
**#1**	Newly-qualified midwives	Community-based and hospital-based rotational posts
**#2**	Team midwives	Community Maternity Unit-based Universal Provision model
**#9**	Senior midwives	Range of senior posts
**#4**	Obstetricians (middle grade to consultant level)	Health Board 1
**#12**	Obstetricians (middle grade to consultant level)	Health Board 2

We exceeded our initial target because we found we could arrange a second group for obstetricians, and because we were able to access women at the local international women’s centre. These were women born outside the UK, although some had been in the UK for many years.

Recruitment of service users did not prove to be problematic; practical difficulties such as childcare and having just given birth were the main identified reasons for non-participation. No practitioner who was approached declined to take part, although, like the service users, some who said they would attend did not turn up on the day. We did not record socio-demographic or professional data concerning the participants: they were recruited solely because of their status as a service user or provider.

The focus groups lasted between 60 and 85 minutes. A note-taker at each focus group recorded details (*e*.*g*. of non-verbal communication, and confirmation of speakers) to help the analysis. Interviews were audio-recorded, transferred to a university password-protected computer, then transcribed by one of the RAs or sent to a professional transcription service. The transcribed interviews and the audio recordings were then stored in a secure cloud-based facility managed by the University of Dundee. Participants were not offered the transcriptions for comment or verification.

In the five service provider focus groups, at least some of the participants were known to at least one of the research team members present. There was no pre-existing relationship between research team members and any of the service user participants.

### Location

The study was conducted in two Scottish health board (HB) areas, chosen because between them they provide care ranging from midwifery-led care (including case-loading for ‘low risk’ women; and team-based community midwifery for women of all risk levels, with intrapartum care for ‘low risk’ women) to ‘high risk’ obstetric care. These models are hosted in different facilities: tertiary units; alongside midwife-led units; a community midwife-led unit located within the grounds of a district general hospital which does not provide obstetric services; and a free-standing midwifery unit.

Eight different venues were used for the twelve focus groups. The group involving women recruited through the international women’s centre was held at that location, while the others were held in meeting rooms in various clinics and hospitals in the two HB areas.

### Ethics

Ethics approval for the study was granted by South Central–Berkshire B Research Ethics Committee (Ref.: 16/SC/0496). We factored in at least one week between the potential participant receiving the invitation letter / PIS and the focus group. The study was discussed as the focus group convened, with an opportunity given for those attending to ask questions. Those willing to proceed signed a standard consent form. All were informed that they had the right to withdraw at any stage, and for any contribution they had made to the discussion to be disregarded. Pseudonyms were given to all participants to help protect their identity.

Due to the terms of our ethical approval and the standard consent form used, supporting data cannot be made openly available. The study protocol is available at the University of Dundee Institutional Repository Discovery: https://doi.org/10.15132/10000135. Further enquiries can be addressed to the lead author.

## Results

The themes and sub-themes that emerged confirmed the feasibility of adapting the QMNC Framework for use as a data collection tool to evaluate stakeholder perspectives of care. Four main themes emerged: ‘Organisation Culture / Work Structure’; ‘Relationships’; ‘Information and support’; and ‘Uncertainty’. All four themes had positive and negative sub-themes; the negative sub-themes from the first three themes created the fourth theme (‘Uncertainty’) (S1 Fig).

In this section we discuss the main themes and how they relate to the Framework. For illustrative purposes we also refer to some of the positive and negative sub-themes which went to make up the themes. A full list of themes and sub-themes is given in Table 2.

**Table 2:** Themes and negative and positive sub-themes mapped against the QMNC Framework.

Themes	Negative sub-themes	Positive sub-themes	QMNC Framework component
**Organisation Culture / Work structure**	**Limited resources / time** * System-driven care * Fear of blame **Critique of the system** * Boundary tensions / roles & responsibilities Work-life balance Apprehension *re* change Inaccessible care Questioning competence	Adequate resources Positive ways of working ^¥^ **Strategies for improvement** * Flexible maternity care ^¥^ Accessible care ^¥^ Competent staff Confidence in the system Effective inter-disciplinary working	Practice categories Organisation of care Values Care providers
**Relationships**	Lack of a relationship Dependant relationship Paternalism	**Positive relationships** * Trust and confidence	Values Care providers
**Information and support** **1**—Communication issues	Poor communication ^¥^ **Lack of / barriers to information** * Poor documentation Poor signposting Inconsistent information	**Effective communication** * Informed women Confidence to speak up Good signposting	Practice categories Organisation of care Values
**Information and support** **2**—Care and management issues	Lack of continuity of care Not involved in care planning **Difficulties with achieving tailored care / lack of tailored care** * Steering women Lack of informed consent Disrespectful care Unrealistic expectations Questioning competence	Strengthening capabilities Continuity of care ^¥^ Autonomy / agency **Tailored care** * Involved in care / sense of control Respectful care Effective inter-disciplinary care Informed consent	Practice categories Organisation of care Values Philosophy Care providers
**Uncertainty**	**Anxiety / confusion** * Muddling through Explaining the system Empowering women ^¥^	**Seeking information and support** * Accepting uncertainty ^¥^ Strengthening capabilities Staff fear	Practice categories Philosophy

* ‘Top 4’ sub-theme for more than one focus group

^¥^ ‘Top 4’ sub-theme for one focus group

The themes arose from discussions of those aspects of the Framework’s characteristics of care which were felt either to work or not work for the individuals concerned. They each mapped back to the Framework, but in a way that shows the interconnectedness of its components (Table 2). For example, ‘Organisation Culture / Work structure’ mapped directly to the Framework’s ‘Organisation of care’ component, but the sub-themes that made up this theme also mapped to ‘Practice categories’, ‘Values’ and ‘Care providers’. Likewise, sub-themes that between them constituted our theme ‘Relationships’ mapped to both ‘Values’ and ‘Care providers’ in the Framework.

Other sub-themes also reflected this inter-connectedness and overlap of the framework categories. For example, ‘Positive ways of working’ (attributed to the theme ‘Organisation Culture / Work structure’) had echoes in ‘Positive relationships’ (Theme: ‘Relationships’) as well as in ‘Continuity of care’ (Theme: ‘Information and support 2 –care and management issues’). ‘Questioning competence’ was a sub-theme for ‘Organisation Culture / Work Structure’ when it related to questioning the way services were organised, but was also a sub-theme for ‘Information and support 2 –care and management issues’ when it related to questioning individual competence.

Illustrative quotes for the most common sub-themes–those that were one of the ‘Top 4’ sub-themes in more than one group—are given in Table 3; these also indicate inter-connectedness.

**Table 3:** Illustrative quotes* highlighting some of the ‘Top 4’ sub-themes that were discussed by more than one group.

Sub-theme	Quote and origin
**Limited resources / time**	Mandy: I think they all need more (booking time) though and with the pressures of health promotion I think you would need to double book every lady, ‘cos if you want to cover smoking, alcohol, (*big sigh from someone else*) feeding choice of for their plan of birth (*Flo*: *yeah*) then you have to do all your documentation, then you audit: have you crossed your t’s and dotted your i’s (*others*: *yeah*) so I think yeah… Interviewer: 40 minutes? Mandy: Yeah, there is always going to be time limits. You can’t squeeze all that in. *[FG2; CMU-based team midwives*, *Universal Provision model]*
**Positive relationships**	Tricia: Yeah, they know you don’t they, they’ve known you now for…from eight weeks to thirty eight weeks, they know you and your husband and they know if you look a little bit more worried from the last time, I just think that’s the continuity, that’s why it’s so good, and also you wouldn’t hesitate to probably say anything, you know like if you were feeling upset you’d say something, whereas if it was just someone that you’ve met at 38 weeks pregnant who you’ve never seen before, you might be a little bit more reluctant to say something, but yeah I think they just know you inside out a little bit better. *[FG7; pregnant women and new mothers*, *case loading model]*
**Lack of / barriers to information**	Eveline: Yeah. The only thing I have missed out on is I didn’t know about I could get massages and stuff so I never ever got the, like, whole thing about it so I missed out, and she said that she was fully booked up until end of March and that’s right before I am due (laughing) so I was like “That’s pointless” (laughing). Serena: I would say the only thing as well is antenatal classes, that’s the only thing that I never knew anything about, until I read the book and seen it on the checklist and I went ‘Ooohhh, maybe I should have asked or known about it (*Eveline*: *mmhhmm*). Because I think, for me, I’m on my own with my husband, so we don’t know what to expect, we don’t know what to do or what to deal with, so you constantly feel like you’re panicking and phoning upstairs and that’s not fair on them (*nervous laugh*). *[FG3; ‘not low risk’ pregnant women]*
**Critique of the system**	Kylie: I can’t say come back in a couple of weeks but oh, actually the first appointment you can get is five weeks’ time. Interviewer: So what’s the answer to that then? Katie: The answer is a central booking line… but we have not got the resources or the clerical or tracking system in place to have that started off. We don’t have any staff for that. We have 130 phone calls a month. We can have that to arrange appointments. That’s not a Band 7 or a Band 6 or a Band 5’s job to do that, I don’t think. I think that’s clerical, but… Interviewer: Is that a commonly held view? Hattie: I think you’re right, you hit the nail on the head there, absolutely, because we’re not doing the job that we’re here to do. *[FG9*: *Senior midwives]*
**Lack of tailored care**	Interviewer: Do you feel like, like at the beginning that health promotion was covered? Fran: Yeah I got that nice piece of information that told me I had a…I was…obese, that nice piece of paper they give you. There you go your BMI is 30 and above… Interviewer: Did it come with information for you to…. Fran: Yes a plate but it’s not very easy when you have Crohn’s when you can’t eat fruit and vegetables. Interviewer: So was it not really adapted for you? Fran: No it was, “Well, we know this will not really work for you, but we have to give it out,” sort of thing. *[FG8; new mothers*, *Universal Provision model]*
**Seeking information and support**	Talia: The first time (I was pregnant) I really wanted that relationship with someone as I wanted to know that everything was ok, but the first midwife wasn’t brilliant she just kept saying “That’s OK, that’s OK, that’s just normal. Everything is normal,” and I just thought “It can’t be…” I was doing a lot of my research and getting a lot of my information on baby centre… Interviewer: Do you think there are people, community is more online is that what you find, rather than having your community….. Talia: Possibly, I think because there is such a wide range of women on there… Whereas this is people from around the world having different experiences, seeing different doctors and midwives and it’s great. They are able to share that with you and it was really helpful for me. It was really supportive to me to know ok I am not alone this is fine *[FG10 (international women’s centre)*, *Universal Provision model]*

* Pseudonyms have been used throughout

### Organisation culture/work structure

This theme links most obviously to the second Framework component, ‘Organisation of care’, but its’ many sub-themes also reflected aspects of the first, third and fifth Framework components (‘Practice categories’, ‘Values’ and ‘Care providers’) (Table 2). For example, the sub-theme ‘Boundary tensions’ reflected wrangles over whether a woman’s complications warranted referral to another practitioner, involving the ‘top right corner’ of the Framework. In essence, this meant disagreements about the indications for midwifery referral to an obstetrician:

“We see… em… a proportion of women for you know, a consultant appointment when actually their issue doesn’t necessarily need to be seen by a consultant… I’ve had women coming just being afraid of certain things about labour and you know, I have actually said to them ‘Have you spoken to your midwife about this?’ and you know, they’ve said ‘No, not really. The midwife just told me to come and see you.’” (FG4:11; Consultant obstetrician #3)

‘Limited resources / time’ was a ‘Top 4’ sub-theme for seven of the groups, including all five of the staff groups. One midwife spoke of being “already stretched, particularly in the community, to complete all our work in the time given” (FG2: 192), while a consultant obstetrician spoke of having to “field the increased workload… It’s resourcing again” (FG4: 278). Other examples are given in Table 3.

### Relationships

The matter of relationships is inherent in personalised health care. While implicit in the Framework components ‘Values’ and ‘Care providers’, the Framework does not explicitly refer to relationships, although its authors note that respectful relationships can strengthen women’s capabilities. In our study, this theme consisted of fewer sub-themes than the other themes, but those that were discussed were obviously crucial, with the significance of positive relationships being stressed by service users and the newly-qualified midwives.

“If we had any worries about anything at all, you could ask (midwife’s name), and if she was concerned about it she would say ‘You need to go upstairs’ or ‘I’ll check’; whatever.” (FG5: 38; Postnatal woman #3)

“Sometimes the really simple things, like a nice couch and the cartoons on for the kids, and when she comes in offering her a glass of water that is not in a plastic cup… or a cup of tea which means ‘You are here, I am listening’. You know, you have some time, it’s not an ‘in-and-out’. They are really, really simple things for me that makes me love that part of the job, ‘cos we are not so clinical and it’s great.” (FG1:202,204; Newly-qualified midwife #1)

These quotes highlight the value of relationships that both women and midwives place on the quality of maternity care.

### Information and support

‘Information and support’ was a distinct theme in that it was partly formed by circumstances which existed before the pregnancy (‘Women’s pre-existing capabilities’; and ‘Community knowledge and help’). Furthermore, the issues that were related to maternity care were divided into two parts: ‘Communication issues’ and ‘Care management issues’. Communication issues were often about reassurance:

“They always ring you back as well, so I think I left a message once and they rang me back the next day, just to answer a few questions that I had because I knew I wasn’t seeing them for about six weeks.” (FG11: 46; Pregnant woman #4)

“I was constantly on the phone, and they were like ‘Don’t worry, it’s fine, you don’t need to worry about it’, but I feel I’ve been constantly on the phone panicking about something, but they’re really good though, they’ve always phoned back.” (FG11: 47; Pregnant woman #2)

Some individual positive sub-themes in ‘Information and support’ reflected specific aspects of the Framework (*e*.*g*. ‘Effective inter-disciplinary care’ is inherent in the Framework’s ‘Care provider’ component, and ‘Tailored care’ is cited as a characteristic of the ‘Values’ component).

“I would say that my midwife from my doctor’s surgery I trust her … and if my bloods were a bit strange she would just call me, like ‘Can you pop down on a Sunday night at 8 o’clock and I’ll just do your blood pressure check’, and that’s really tailored and that makes me put a lot of faith in that she knows what’s going on, and actually thinking about my care throughout the week, not just when I’m in the actual surgery or whatever.” (FG6: 231; Pregnant woman #1)

There are clear links between this sub-theme and the preceding one on relationships. We have referred to the notion of ‘information and support’ which results in reassurance and a feeling of trust.

### Uncertainty

The theme ‘Uncertainty’, arising from the other themes’ negative sub-themes, was an unexpected finding. Its emerging sub-themes could often be traced back to the original negative trigger. For example, some women who experienced ‘Lack of or barriers to information’ responded to the resulting uncertainty by ‘Seeking information and support’; this was effectively a loop-back mechanism to address a perceived problem. However, this approach did not necessarily relate to the model of care experienced: in FG10, women born outside the UK, having encountered a lack of relevant information when attending the midwife or obstetrician, related how they sought to remedy this from online sources such as support groups. However, not all uncertainty resulted in positive reactions. Whether ‘Muddling through’ is even acceptable may be open to debate, but for pregnant women and new mothers to report becoming anxious and confused is concerning. For practitioners to indicate that some became fearful as a response to uncertainty is also a cause for concern.

More positively, obstetricians also related that they spent a good deal of time explaining things to pregnant women:

“There is a big immigration population at the moment … Every single consultation, we spend fifty percent of the time explaining to her, ‘This is the way we do things here’.” (FG12: 47; Consultant obstetrician #5).

“We often use the phrase ‘guidelines are guidelines’ and care has to be individualised as (#5) said … we’re quite comfortable explaining that to patients, but you know, maybe other people don’t quite understand that… you don’t have to follow it to the T if it’s not appropriate…” (FG4: 106; Consultant obstetrician #3).

## Discussion

Our principal aim in this study was to assess the feasibility of developing the QMNC Framework into an evaluation toolkit through conducting focus groups with some of the principal stakeholders in maternity care. The Framework is a high-level instrument which has already been used by the World Health Organisation to develop antenatal care guidelines [11], by the Scottish Government to develop maternal and newborn care policy [2], and has been proposed as a means by which global midwifery standards can be improved [9]. However, to our knowledge this is the first time it has been used as a data collection tool to benchmark care quality.

Our intention in this paper is not to provide a detailed analysis of the themes and sub-themes that emerged from our analysis of the twelve focus groups; rather it is to assess how well these can be mapped back to the QMNC Framework, and whether this provides a solid basis for pursuing our long-term goal of developing an evaluation toolkit. As such, our first step was to identify the aspects of care that were most important or salient to different stakeholders. While we have noted how different participant groups contributed to the development of the sub-themes (and thereafter to the main themes), this is merely to indicate in this paper the inclusive nature of our analysis. In a separate paper we report a comparative analysis of sub-themes raised by those with experience of or expertise in different models of care.

Of our four identified main themes, only ‘Organisation Culture / Work Structure’ mapped directly to a single Framework component of care. As illustrated by the quote in the results section there was some disagreement about the referral process. Renfrew et al [8] note the importance of having appropriate referral mechanisms between professional groups and services, but with increasing complexity in people’s lives this is evidently something that causes inter-disciplinary friction in maternity care. Proposed ‘community hubs’ that allow for more effective interaction between health care and social care services, are advocated in both *Better Births* [1] and *The Best Start* [2]. Limited resources were also noted as a top four subtheme. Limited resources, and especially limited time, may impact adversely on other features of care [13]. Pressure of time in a busy clinic militates against effective communication and thereby reduces the opportunity to develop a trusting relationship.

Both our ‘Relationships’ and ‘Information and support’ themes illustrate well the interconnectedness of the Framework’s components, as did the emergence of ‘Uncertainty’, which arose from the other themes’ negative sub-themes. For example, ‘Information and support’ clearly relates to ‘Relationships’, because communication is an essential component of care. We have referred to the notion of ‘information and support’ resulting in reassurance and a feeling of trust. This echoes McCourt and Stevens’ [14] findings from a large study of caseload midwifery about information being more in the form of dialogue than a one-way process, and the feeling of personal control which results from this. Indeed, while communication is specified in the Framework’s ‘Values’ component of care, it runs implicitly through all five components. As indicated in Table 2, we found that there was considerable inter-connectedness in the Framework. This applied to both the positive and negative reports of care.

Identifying what works well is important. The link between continuity of care / continuity of carer and positive relationships is well-recognised [15, 16]. It may seem intuitive that a positive relationship will help improve outcomes. Dahlberg and Aune’s qualitative study in Norway [17] found that positive relational continuity was highly prized by women. However, they concede that while clinical outcomes are easily assessed, measuring these relationships–and thereby identifying possible evidence for the underlying mechanism—is difficult, a challenge also noted by Kennedy et al [18].

It is also essential to identify the negative aspects of care so that these can be addressed. We found that some of these negative aspects mirrored positive Framework characteristics of care, (e.g. ‘Lack of tailored care’ is obviously the reverse of the Framework’s ‘Care tailored towards women’s circumstances and needs’). The Framework, in highlighting the components and characteristics of care that result in the best outcomes, is framed in positive terms. While noting that the absence of these features militates against good outcomes, it does not focus on the process or mechanisms that result in those poorer outcomes. Our derivation of the theme ‘Uncertainty’ underlines the importance of identifying and addressing negative aspects of care. Diamond-Brown [19], in his study of obstetricians in the USA, has noted the importance of continuity of care in navigating the difficulties of uncertainty. He asserts that ‘patient-centred decision-making’ is hampered when care is fragmented, and this in turn can lead to poorer outcomes.

Not all negative sub-themes could be linked directly to the Framework, although there was an inherent connection. For example, ‘work-life balance’, which we related to the theme ‘Organisation culture / work structure’, and which is an important feature of contemporary health care, is not explicitly a feature of quality care as determined by the Framework. The literature would suggest that having a good work-life balance is necessary if practitioners are to provide the highest quality care [20]. Clearly, the Framework cannot reflect all experiences of care; policy makers and managers need to be aware of the organisational requirements underpinning the delivery of quality care.

The largely successful mapping of the sub-themes back to the QMNC Framework suggests that the Framework, sensitively adapted, can be a useful exploratory data collection tool in its own right, and is a useful highlighter of areas that require more detailed and more comprehensive evaluation. This will be of value to service planners, as it provides evidence of local contextual issues that may need to be accommodated.

We acknowledge that there is a certain circularity to our approach: having started with the Framework, we asked open questions based on it, and then were able to map responses back to the Framework. We offer two responses to this criticism: firstly, any exploration of perceptions of experiences about the quality of care would do well to base itself on the most comprehensive description to date of what quality care should comprise. Secondly, our finding that the sub-themes tended to map to multiple Framework components of care rather than a single component shows how the Framework components are inter-related. The care experience is complex and unsuited to simple classification. If we are to identify why and how the good models work, we must be prepared to engage with this complexity. The significance of relational issues, which appear to be key to this complexity, have long been recognised: Hunter et al [21] note the wealth of evidence demonstrating the significance of the woman-care giver relationship.

We re-phrased some questions based on the experience of facilitating the discussions. This included the opening question, which was originally “What do you feel about the organisation of care?–e.g. is/was it accessible, of good quality, and adequately resourced?” (*pace* the Framework). We subsequently changed this for service users to “How do you feel care is organised–is it flexible, able to fit in with your family’s needs?”, and for service providers to “Do you think that you have accessible care that’s available to women, that they know how to access in order to get the right care providers?” While the Framework is so detailed that it would not be possible to include questions covering every characteristic of care contained therein, by using an adaptive approach to the focus group topic guides, we were able to refine our line of questioning, which we believe allowed us to cover the Framework reasonably comprehensively. The Lancet Series authors have noted that the Framework is not set in stone: as new evidence emerges about what constitutes good quality care, it will need to be amended.

The next planned phase of this programme of research is to refine this approach in three different settings: in an evaluation of maternity care in Australia, in an assessment of service user perceptions in different models of care in the Netherlands, and in a midwifery continuity of carer scheme in Scotland which is aimed at promoting home birth. Thereafter we plan to extend this to a wider evaluation of maternity care in Scotland as *The Best Start* [2] is implemented. In that planned phase of the programme of research we will assess the extent and nature of community-based midwifery continuity of carer provision in Scotland in both higher risk and lower risk populations, and identify the facilitators and barriers which influence the implementation or extension of community-based midwifery continuity of carer models.

### Limitations

We acknowledge that our study has a number of limitations, and we are not claiming to have established anything conclusively. Our intention was to explore whether the Framework could feasibly be adapted to explore the experience of maternity care. Further focus groups with different people may highlight different issues. We acknowledge that other groups of health care workers (such as paediatricians, maternity care assistants and administrative staff) and service users (such as the women’s partners or other family members) might provide different insights. The study areas included do not have as great an ethnic diversity as many other areas in the UK and we tried to off-set this by having one focus group (FG10) for woman who were all born outside the UK. Further, although we advertised in clinic areas and staff areas so that interested parties could contact the research team and ask to take part, those doing so may not be typical.

One non-participant (a health professional) was unexpectedly present for some of FG8. As she was a care-giver for most of the women present there is a possibility that her presence may have encouraged social desirability bias. However, as this was the most negative in tone of all the focus groups, we do not feel this was the case. The focus group facilitators are all experienced midwives, and all experienced qualitative researchers. The broad range of both positive and negative comments suggests that the participants, including those service providers who were previously known to the researchers, were not trying to give socially or professionally desirable answers.

Gender may have played a part in conducting some of the focus groups. AS (the only male member of the research team) facilitated FG10 (Senior midwives), some of whom he knew. MW and KF facilitated the focus groups with obstetricians, all of whom they knew and some of whom were male.

### Conclusion

This was an exploratory study assessing the feasibility of adapting the QMNC Framework for the purpose of assessing the extent to which good quality care is perceived to be present in local maternity services. We established that this was indeed feasible. The Framework is designed to reflect quality care; suitably worded open questions based on its components and characteristics of care allowed participants to identify a wide range of issues that were clearly of relevance or concern. While the Framework lists characteristics of care under specified components of care, we found that the care experience of these diverse stakeholders, as identified by the emerging sub-themes, frequently crossed these components’ boundaries, showing the Framework’s inter-connectedness.

We involved pregnant women, new mothers and midwives who had experience of different models of hospital-based and community-based maternity care, as well as obstetricians. All had a direct interest in seeing good quality care in practice. The range of participants, while not comprehensive, was broad enough to raise both expected and unexpected sub-themes, highlighting both what is working well and what is felt not to work well in local maternity services. In our next papers we will report the areas of commonality and difference between different groups with experience of different models of care.

Although this was exploratory research in two localities, it was large for a pilot study, consisting of twelve focus groups comprising 69 participants. We are encouraged by this initial phase of our planned programme to develop an evaluation toolkit that can be applied to maternity services in a wide range of settings; further developmental work is underway.

## Supporting information

S1 File Focus group topic guide. (PDF)

S1 Fig Interaction of emerging themes. (TIF)
