# Supplementary material for: Adapting the Quality Maternal and Newborn Care (QMNC) Framework to evaluate models of antenatal care: A pilot study
Source: PLoS One. 2018 Aug 14;13(8):e0200640. doi: 10.1371/journal.pone.0200640 (PMC6091915; doi:10.1371/journal.pone.0200640)
Supplement: S1 File — (PDF) [file pone.0200640.s001.pdf]

Topic guide for focus groups (service users and service providers)

These questions * relate to the model of care with which you are most familiar

- Did the model of care cover all the necessary bases – e.g. health promotion, screening, care planning, managing complications?
  - E.g., does / did it provide the necessary care for woman whatever their risk level?

- What do you feel about the organisation of care?
  - E.g., is / was it accessible, of good quality, and adequately resourced?

- Do you feel that women are shown respect within this model of care?
  - E.g., is / was care tailored to the needs of individuals?

- Is / was the care based on a promoting normality and strengthening women's capabilities?
  - E.g., does / did it follow expectant management, intervening only when necessary?

- Do / did care providers demonstrate both knowledge and skills, and an awareness of how to use these most effectively?
  - E.g., are roles and responsibilities based on need? On resources?

** questions are based on the QMNC Framework.*
Present tense to be used for pregnant women and service providers; past tense to be used for postnatal women.
