# Supplementary figures and images for: Adapting the Quality Maternal and Newborn Care (QMNC) Framework to evaluate models of antenatal care: A pilot study
Source: PLoS One. 2018 Aug 14;13(8):e0200640. doi: 10.1371/journal.pone.0200640 (PMC6091915; doi:10.1371/journal.pone.0200640)

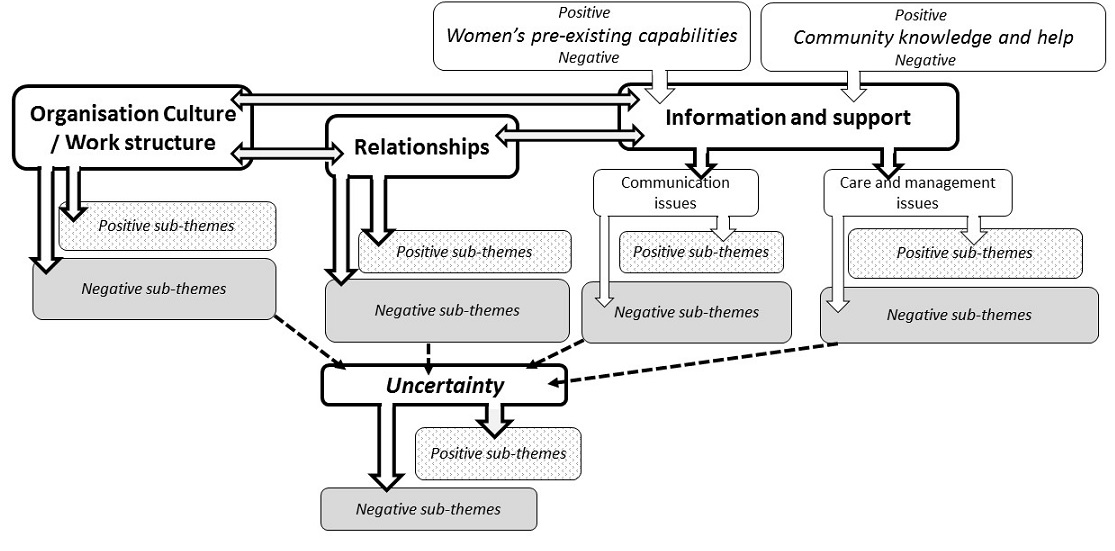

Supplement: S1 Fig — (TIF) [file pone.0200640.s002.tif]
